# Use of multimodality imaging, histology, and treatment feasibility to characterize a transgenic *Rag2*-null rat model of glioblastoma

**DOI:** 10.3389/fonc.2022.939260

**Published:** 2022-11-22

**Authors:** Luke R. Jackson, Megan R. Masi, Bryce M. Selman, George E. Sandusky, Hamideh Zarrinmayeh, Sudip K. Das, Surendra Maharjan, Nian Wang, Qi-Huang Zheng, Karen E. Pollok, Scott E. Snyder, Phillip Zhe Sun, Gary D. Hutchins, Elizabeth R. Butch, Michael C. Veronesi

**Affiliations:** ^1^ Department of Radiology and Imaging Sciences, Indiana University (IU) School of Medicine, Indianapolis, IN, United States; ^2^ Department of Pathology and Laboratory Medicine, Indiana University (IU) School of Medicine, Indianapolis, IN, United States; ^3^ Department of Pharmaceutical Sciences, Butler University, Indianapolis, IN, United States; ^4^ Department of Pediatrics, Indiana University (IU) School of Medicine, Indianapolis, IN, United States; ^5^ Department of Radiology and Imaging Sciences, Emory School of Medicine, Atlanta, GA, United States

**Keywords:** glioblastoma, magnetic resonance imaging (MRI), positron emission tomography (PET), hybrid PET/MRI, amino acid PET, CEST MRI, amide proton transfer (APT) imaging

## Abstract

Many drugs that show potential in animal models of glioblastoma (GBM) fail to translate to the clinic, contributing to a paucity of new therapeutic options. In addition, animal model development often includes histologic assessment, but multiparametric/multimodality imaging is rarely included despite increasing utilization in patient cancer management. This study developed an intracranial recurrent, drug-resistant, human-derived glioblastoma tumor in Sprague–Dawley *Rag2*-*Rag2* ^tm1Hera^ knockout rat and was characterized both histologically and using multiparametric/multimodality neuroimaging. Hybrid ^18^F-fluoroethyltyrosine positron emission tomography and magnetic resonance imaging, including chemical exchange saturation transfer (^18^F-FET PET/CEST MRI), was performed for full tumor viability determination and characterization. Histological analysis demonstrated human-like GBM features of the intracranially implanted tumor, with rapid tumor cell proliferation (Ki67 positivity: 30.5 ± 7.8%) and neovascular heterogeneity (von Willebrand factor VIII:1.8 to 5.0% positivity). Early serial MRI followed by simultaneous ^18^F-FET PET/CEST MRI demonstrated consistent, predictable tumor growth, with exponential tumor growth most evident between days 35 and 49 post-implantation. In a second, larger cohort of rats, ^18^F-FET PET/CEST MRI was performed in mature tumors (day 49 post-implantation) for biomarker determination, followed by evaluation of single and combination therapy as part of the model development and validation. The mean percentage of the injected dose per mL of ^18^F-FET PET correlated with the mean %CEST (r = 0.67, P < 0.05), but there was also a qualitative difference in hot spot location within the tumor, indicating complementary information regarding the tumor cell demand for amino acids and tumor intracellular mobile phase protein levels. Finally, the use of this glioblastoma animal model for therapy assessment was validated by its increased overall survival after treatment with combination therapy (temozolomide and idasanutlin) (P < 0.001). Our findings hold promise for a more accurate tumor viability determination and novel therapy assessment *in vivo* in a recently developed, reproducible, intracranial, PDX GBM.

## 1 Introduction

### 1.1 The disease

Glioblastoma (GBM) is a highly malignant brain tumor with a poor prognosis (approximately 20 months) despite the aggressive standards of care (1). The latter includes surgery, radiation, temozolomide (TMZ), and tumor-treating fields (2). GBM is notoriously resistant to treatments secondary to a high propensity for mutations, cellular heterogeneity, high proliferative capacity, aggressive angioinvasive properties, and resistance to apoptosis (3–6). Among the various histologic characteristics that allow a diagnosis of GBM, microvascular proliferation and central necrosis remain as key delineators when there is diffuse astrocytic morphology in the latest 2021 World Health Organization Classification system update (7). While not a required diagnostic entity for GBM diagnosis, the percent Ki67 proliferation or Ki67 Index is often included in glioma tissue analysis (8). Ki-67 is a nonhistone nuclear protein associated with ribonucleic acid (RNA), is present in higher amounts in cells entering the mitotic cycle, and is widely used to measure cellular proliferation in the assessment of gliomas, including GBM (9). Therefore, the characteristics of microvascular proliferation/neovascularity and Ki67 index were also measured in this study during animal GBM model development.

### 1.2 The GBM cell model

An important goal of GBM model development is to work with GBM cell lines that more closely represent the resistant behavior of GBM in patients. The chosen tumor cells to implant must be selected and developed carefully to maintain common hallmarks of the primary tumor, such as mutations and amplifications, which can be lost through cell passage over time. For example, immortalized cell lines such as U87, T98G, and U251 are prone to genetic drift and, when grown heterotopically, have shown difficulty mimicking the tumor microenvironment (10). This has also been shown to occur in rat glioma cell lines (9L, C6, and F98) (11). In contrast, xenografts derived from patients (PDX), when grown in immunocompromised rodents, may overcome these limitations by maintaining genetic and histologic similarity to human GBM (10, 12). For instance, the Mayo Clinic GBM Xenograft National Resource has thoroughly characterized numerous patient-derived xenografts (PDX) using previously described methods (13). The GBM10 cell line from this resource and utilized in this study has wild-type p53, epidermal growth factor (*EGFR*) amplification, an unmethylated O6-methylguanine-DNA methyltransferase (*MGMT*) promoter, and wild-type isocitrate dehydrogenase (*IDH*) (13). These characteristics represent at least one subset of aggressive GBM tumors with a poor prognosis (14). However, a recent study demonstrated that using magnetic resonance imaging (MRI)-based diffusion and perfusion-weighted techniques following PDX growth of certain tumors intracranially was not able to recapitulate the original MRI features found in the corresponding patients the tumors were derived from (15). Therefore, PDX models can benefit from advanced imaging to help with studying the maintenance of the original tumor properties.

### 1.3 The GBM animal model

Animal models, most commonly involving rats and mice, allow important information to be gathered for translational biomedical research because of their similar anatomy and physiology to those of humans despite their small size (16). Since the development of the first transgenic mouse model in 1981, mice have since been extensively used in biological research due to their ease of genetic modification, higher throughput efficiency, and lower cost relative to rats (17). However, compared to mice, rats have greater physiological similarities to humans, which allows for a more accurate translation of preclinical pharmacokinetics and pharmacodynamics to the clinic (18). In addition, the rat is eight to ten times larger than the mouse, with a higher brain volume, presenting several practical advantages (19). For instance, the large rat brain is more favorable for cross-sectional imaging assessment. Given the need for a more reliable immunodeficient rat model for human xenograft placement, Noto et al. (20) developed a recombination activating gene 2 (*Rag2*) knockout mutation (*Rag2*-null) in Sprague–Dawley (SD) rats, rendering them devoid of B- and T-cells (20). They verified that a flank-implanted GBM xenograft (U87) could grow in several models, including the *Rag2*-null SD model. However, intracranially implanted tumors using this new rat strain with the incorporation of histologic and multi-modality neuroimaging features have not yet been detailed.

### 1.4 GBM imaging

Conventional MRI is a widely used modality, given its greater ability to distinguish tissue abnormalities on cross-sectional imaging. Still, it can lack specificity regarding tumor presence without more advanced MR imaging methods. GBM tumors can demonstrate infiltrative mass-like appearance on fluid-attenuated inversion recovery (FLAIR), increased contrast enhancement following administration of gadolinium contrast, and increased diffusion restriction on diffusion-weighted Imaging (DWI) (21). The issue of peri-tumoral edema on T2-weighted sequences (which includes FLAIR) in animal models was highlighted by 11 since using a T2 sequence cannot separate the tumor from the edema (11). Thus, they demonstrated the advantage of using chemical exchange saturation transfer (CEST) MRI, which detects mobile proteins and peptides as a molecular marker for cellularity without the need for the exogenous administration of gadolinium contrast (22–25). This technique has since shown great promise for detecting a tumor in humans for malignant brain tumors and is rapidly moving towards clinical use. Other multiparametric MR imaging techniques used in animal imaging to delineate GBM tumors from background non-specific tissue abnormalities, including following treatment, are MR Spectroscopy (26, 27) and MR Perfusion (28), among others.

During PET, uptake of various radiotracers through membrane channels or receptor binding in tumor tissue greater than normal brain tissue allows increased specificity of tumor presence when conventional MRI is otherwise nonspecific. 2-^18^F-fluoroethyl-*L*-tyrosine (^18^F-FET) is an effective amino acid radiotracer that is used clinically and is passively taken into cells *via* the system L amino acid transporter (LAT) in exchange for leucine (29–31). LAT receptors are overexpressed in brain tumor cells undergoing rapid proliferation during tumor angiogenesis (32, 33). A recent clinical study using ^18^F-FET PET showed an excellent ability to delineate the location of viable tumors from underlying nonspecific treatment changes with an accuracy of 96% (100% sensitivity and 91% specificity) (34). In rodents, 35 detailed a novel methodology using PET to more accurately measure brain tumor volumes following up take of ^18^F-FET in a mouse model (35).

While there are few examples of multiparametric, multimodality imaging utilizing PET and CEST MRI in animal models of GBM, there are other examples of the use of tumor-specific PET targeting agents combined with multiparametric MRI for increased diagnostic accuracy. For example, ^18^F-DPA-714 is a radiotracer targeting the 18 kDa translocator protein (TSPO), comparable to ^18^F-FET PET for detecting tumors when trialed in a mouse model of infiltrative human glioma (36). Furthermore, a quantitative form of Diffusion-weighted MRI known as diffusion kurtosis was also performed. The authors proposed that ^18^F-DPA-714 and diffusion kurtosis may be an improvement over standard imaging methods to visualize early glioma growth and infiltration. In addition, Kim et al. demonstrated the superiority of ^18^F-FET PET for therapy assessment and prognostication compared with bioluminescence (BLI) or MRI when evaluating an antiangiogenic drug, bevacizumab, in an intracranial U87 GBM Nude mouse model (37). In mice with intracranial U251 GBM implants, PET and MRI were used to test the potential of poly(ADP-ribose) polymerase (PARP)-targeted PET radiotracer ^18^F-PARPi, which discriminated radiation injury from tumor better than ^18^F-FET in the contrast-enhanced portion of the brain (38).

Although we could not locate a published preclinical study utilizing a combination of ^18^F-FET/CEST MRI, this multimodality approach has already shown tremendous potential for clinical use in therapy response assessment. For instance, the acquisition of simultaneous CEST MRI with ^18^F-FET for assessment of the progression of disease versus treatment-related change in GBM patients yielded a diagnostic accuracy of 86% (39). The most striking part of these findings was that the imaging analysis was fully automated using machine learning techniques. Of major interest for biomarker determination is the complementary and possibly synergistic information gleaned from hot spot volumetric delineation in tumors using multiparametric MRI imaging or when comparing ^18^F-FET PET with MRI. Da Silva et al., 2018 compared hot spots derived from 3D volumetric assessment ^18^F-FET PET and CEST MRI in eight patients with gliomas, showed an absence of correlation between the two modalities with an average distance of 20 ± 13 mm between CEST and ^18^F-FET hot spots (40). The authors suggested both modalities could be used as biomarkers to better study the increasingly important issue of glioma heterogeneity. A subsequent clinical study in a larger number of patients showed a relevant spatial overlap between glioma hotspot volumes using ^18^F-FET and CEST MRI in enhancing and non-enhancing FLAIR abnormal tissue (41). In this study, the ^18^F-FET did not completely overlap with the CEST hot spots.

### 1.5 GBM therapy

TMZ is the only first-line, the standard of care chemotherapy agent showing significant efficacy against GBM (42). However, during treatment, GBM often acquires TMZ resistance (43). Since TMZ is the only first-line standard-of-care chemotherapy agent for GBM, drug studies often include it for comparison. TMZ nonspecifically alkylates DNA, inducing a rapid increase in the normal p53 tumor suppressor protein, activating DNA repair, cell cycle arrest, or apoptosis (44). However, the p53–mouse double minute 2 (MDM2) pathway is deregulated in up to 84% of GBM patients (45). When p53 is dysfunctional, apoptosis is circumvented, and TMZ resistance increases (46–48). Thus, clinical cases of GBM often recur in the first 1–2 years of initial treatment (49, 50). One rationale for overcoming TMZ resistance is to target the p53–MDM2 cascade by inhibiting MDM2, an important negative regulator of p53. Idasanutlin is a second-generation MDM2 inhibitor with higher potency and blood-brain barrier (BBB) penetration than first-generation nutlins. It is currently being tested in various cancer clinical trials, including GBM (NCT03158389) (51).

### 1.6 Purpose

This study aimed to develop a recurrent, drug-resistant patient-derived tumor model (GBM10) using the novel *Rag2*-null rat and to determine whether the tumor maintains the histologic/pathologic features of GBM in humans. Advanced neuroimaging was performed for *in vivo* anatomical and molecular characterization using simultaneous ^18^F-FET PET/CEST MRI. Moreover, we assessed therapy feasibility using single and combination oral treatments during animal model development. These results also help address the critical need for developing animal models that more closely mimic the resistance properties of human GBM.

## 2 Materials and methods

### 2.1 Cell culture

All cell culture experiments were conducted with approval from our Institutional Biosafety Committee (IBC Protocol #IN-984). GBM10 cells (via material transfer agreement with the Mayo Clinic and initial courtesy of the IU Simon Cancer Center *In vitro* Therapeutics Core, Dir. Dr. Karen Pollok) were thawed and then grown in Dulbecco’s Modified Eagle Medium (Life Technologies Corporation, Grand Island, NY, USA) containing high glucose concentration, L-glutamine, and HEPES, and supplemented with 10% fetal bovine serum. All cells underwent short-term culture (7–14 days) at 37°C in a 5% CO_2_ incubator.

### 2.2 Intracranial GBM10 implantation

Eight-week-old *Rag2*-null rats were obtained from Envigo (Indianapolis, IN, USA) and acclimated for 1 week. On the morning of the surgical intracranial implantation of GBM10 cells, all rats received a subcutaneous injection of Ethiqa XR (0.65 mg/kg) to relieve postoperative pain and discomfort. Each rat was anesthetized using 5% isoflurane (5 L oxygen/min) and maintained under 2% isoflurane (2 L oxygen/min). Once deeply anesthetized, based on the absence of the toe pinch reflex, an ocular ointment was placed over the animal’s eyes, and the animal’s head was shaved. The animal was then placed onto a sterilized stereotactic frame (Harvard Apparatus, Holliston, MA, USA) with an underlying heating pad set at 37°C. All procedure steps were performed under sterile conditions. Respiration was also monitored during the entire procedure. With the head placed securely in the ear bars and following standard sterilization, an anterior-posterior midline incision was made along the cranium, and the scalp and periosteum were bluntly dissected to expose the cranial sutures. The bregma was identified at the junction of the superior sagittal and coronal sutures. Using a sterile drill with a 1.4 mm bit and attached to the stereotactic unit, a hole was drilled into the skull 3 mm lateral and 1 mm anterior to the bregma. Each rat was injected with 30,000 cells in 5 µL phosphate-buffered saline (PBS) using a stereotactically guided microsyringe (700 Series, Hamilton, Franklin, MA, USA) affixed with a 27-gauge needle. The needle was inserted 5.5 mm deep from the outer table of the cranium into the striatum. The cells were injected at a rate of 2 µL/min. After ensuring an absence of reflux for 5 min, the needle tip was slowly removed at a rate of 0.6 mm/min, the burr hole was sealed with sterile bone wax, and the incision site was sutured using 4-0 nylon sutures. Triple antibiotic ointment was then applied to the incision, and the animals were placed in their cages to recover on a warm blanket. The rats were observed once daily for 4 days and supplemented with wet feed to ensure full recovery and twice weekly thereafter until treatment.

All animal procedures and experiments were conducted following the guidelines and regulations set forth by the Institutional Animal Care and Use Committee of the Indiana University School of Medicine (Animal Use Protocol: 20118).

### 2.3 Imaging

All MRI experiments were performed on a 30 cm bore 9.4T magnet (BioSpec 94/30, Bruker, Billerica, MA, USA) with a PET insert. Images were acquired using a 72 mm quadrature volume resonator as the transmitter, and a four-element (2 × 2) phased array coil as the receiver. Before each imaging session, all animals were anesthetized using isoflurane, and an indwelling tail vein catheter was placed to enable injection of the MRI contrast agent gadobenate dimeglumine (Magnevist, Bayer Whippany, NJ, USA) and the radiotracer ^18^F-FET.

All rats underwent early MRI screening after GBM implantation at days 21 and day 35, which included 2D T2 weighted MRI to determine if a lesion was present and increasing in size. The 2D T2-weighted anatomical images were obtained using a standard 2D rapid acquisition with refocused echoes (RARE) sequence with a ratio of repetition time (TR) to time-to-echo (TE) TR/TE = 3000 ms/22 ms and RARE factor = 8. The matrix size was 256 × 256, the field of view (FOV) was 32 mm × 32 mm, and the resolution was 125 µm x 125 µm. The scan time was 6 min 24 s.

For subsequent multimodality imaging, MRI included a 3D T1 post-contrast sequence, 3D T2-weighted sequence, and CEST MRI sequence, performed simultaneously to ^18^F-FET PET. The same protocol was followed for phase 1 (N=5, days 42 and 49) and phase 2 (N=15, day 49). At the start of imaging, the animals were injected with gadobenate dimeglumine and ^18^F-FET (100 uL bolus of 14.6 ± 1.4 MBq) in a tail vein catheter. The MRI sequence order is as follows: 3D T1-FLASH post-contrast imaging was performed immediately after gadolinium injection at the beginning of the imaging session for 19 min. The 3D T2-weighted sequence was then acquired over 20 minutes. The CEST MRI sequence was then acquired for the final 17 mins. PET imaging began 20 minutes following injection, with a collection of static imaging from 20-40 minutes after injection.

The 3D T1-weighted post-contrast anatomical images were obtained using a 3D fast low-angle shot (FLASH) sequence with TR/TE = 50 ms/8.4 ms after contrast agent injection. The matrix size was 320 × 320 × 64, the FOV was 35 mm × 35 mm × 16 mm, and the resolution was 110 x µm 110 x µm 250 µm. The scan time was 19 min. 3D T2-weighted anatomical images were obtained using a standard 3D RARE sequence with TR/TE = 1550 ms/32 ms and RARE factor = 8. The matrix size was 120 × 120 × 48, the FOV was 30 mm × 30 mm × 12 mm, and the spatial resolution was 250 µm x 250 µm x 250 µm. The scan time was ~20 min. The CEST experiments were performed using a 2D RARE sequence with TR/TE = 3000 ms/18 ms and RARE factor = 10. The saturation pulse was 1 second with an amplitude of 5 µT, and the saturation offset sweeps ranged from –5 ppm to 5 ppm, with 0.25 ppm increments. The matrix size was 128 × 128, and the FOV was 32 mm × 32 mm. The slice thickness was 2 mm, and the scan time was 17 min. A water saturation shift referencing method was applied to correct the B_0_ map (52). The saturation pulse amplitude was 0.5 µT, and the saturation offset sweeps ranged from –1.5 ppm to 1.5 ppm, with 0.125 ppm increments for water saturation shift referencing. The scan time was 10 min. The MTR_aysm_ and amide-CEST at 3.5 ppm maps were calculated after B_0_ correction using MATLAB (MathWorks, Natick, MA, USA) (25).

High molar activity ^18^F-FET was prepared at the Indiana University PET Radiochemistry Facility by reacting anhydrous ^18^F-fluoride with *O*-(2-tosyloxyethyl)-*N*-trityl-*L*-tyrosine *tert*-butylester (TET) (ABX Advanced Biomedical Compounds, Radeberg, Germany) using slightly modified established methods (53–56). Product release criteria were in accordance with ^18^F-FET prepared for human use (IND 150883, Veronesi). The product radiochemical purity was always ≥99%. The emission recording for the interval was acquired at 20–40 min post-injection. The image reconstruction procedure was based on the maximum a posteriori (MAP) 0.25 mm algorithm with 24 iterations. Registration of PET images to 3D anatomical MRI was performed using the PMOD fusion tool (PMOD Technologies Ltd., Bruker, Zurich, Switzerland).

### 2.4 Imaging analysis

Tumor volumetric and cross-sectional analyses were performed using the imaging software MIM7 (MIM Software Inc., Cleveland, OH, USA). The tumor boundaries were manually contoured for MRI and PET analyses based on the observed signal hyperintensity present on T2-weighted images and/or contrast-enhanced regions on post-Gd T1-weighted images.

First, a subset of rats (n = 5) underwent serial ^18^F-FET PET/CEST MRI on days 42 and 49. ^18^F-FET radiotracer uptake was quantified as the maximum and mean percentage of the injected dose per mL (%ID/cc max and (%ID/cc mean). The volumes of interest of the tumor and mirrored contralateral control region were determined, and the maximum and mean tumor-to-brain ratio (TBRmax and TBRmean) were quantified based on ^18^F-FET PET activity. These metrics are recommended by the international PET working groups European Association of Neuro-Oncology (EANO)/Response Assessment for Neuro-Oncology (RANO). The volume of interest (VOI) was obtained by semi-automated analysis by first finding the area of maximum ^18^F-FET uptake and then including within the VOI all activity that reached at least 25% of the maximum activity. For CEST MRI measurements, a region of interest was manually drawn around the visibly hyperintense signal on T2 sequences and a mirrored area within the contralateral normal brain. The Z-spectrum, MTR_asym_, and amide-CEST MRI maps of GBM10 tumor-bearing rat brains were evaluated. The mean %amide-CEST MRI (Mean %CEST) and mean TBR of %CEST MRI (Mean %CEST TBR) were recorded for each animal. Multiparametric fusions of representative rats were performed using PMOD v4.2 (PMOD Technologies, Zurich Switzerland) by importing DICOM files of 3DT1-FLASH Post-Contrast, ^18^F-FET PET, and CEST MRI, and overlaid for visual assessment and interpretation of hot spot delineation.

For the larger cohort of rats (n = 15) imaged in phase two, a correlation study was performed at day 49 post-implantation regarding the capacity for^18^F-FET PET and CEST MRI to provide complementary molecular information for quantitative imaging biomarker determination. The imaging parameters, including Max %ID/cc, Mean %ID/cc, TBRmax, TBRmean, Mean %CEST, and Mean %CEST TBR, were assessed in the tumor and control tissues of each animal.

### 2.5 Therapy administration

Once tumor characterization was completed for all 15 rats in phase two, they were separated into three treatment groups: vehicle-treated group (control; n = 5), TMZ-treated group (n = 5), and TMZ + idasanutlin-treated group (n = 5). For drug preparation, a PBS solution was first adjusted to pH ≤ 3 using anhydrous citric acid (Sigma-Aldrich, Burlington, MA, USA). Powdered TMZ (Sigma-Aldrich, Burlington, MA) was diluted to 6.6 mg/mL concentration in 20–25 mL acidified PBS. For the combination treatment, TMZ and idasanutlin (Med Chem Express, Monmouth Junction, NJ, USA) were diluted to a concentration of 6.6 and 5.0 mg/mL, respectively, in 20–25 mL acidified PBS. This solution was composed of up to 10% dimethyl sulfoxide (DMSO) (v/v) to aid in the dissolution of both chemotherapeutic compounds. The control solution consisted of pH 3 anhydrous citric acid and DMSO in PBS. In the treatment groups, each animal received a dose of TMZ (66 mg/kg) either alone or with idasanutlin (50 mg/kg). The animals were treated thrice weekly *via* oral gavage until near-death endpoint criteria were reached based on clinical observations. At this point, the animals were euthanized *via* transcardiac perfusion and were included in an overall survival analysis using the Kaplan–Meier method. For near-death endpoint criteria assessment, a behavior point system was applied to each animal as previously described (57).

### 2.6 Transcardiac perfusion

Once near-death endpoint criteria were reached, the animals were euthanized *via* transcardiac perfusion under full anesthesia. Animals were anesthetized using 5% isoflurane (2 L oxygen/min), placed on a dissection pan, and maintained under mask anesthesia in a supine position using 2% isoflurane (2 L oxygen/min). A transverse incision was made through the skin on the abdomen, and the peritoneal cavity was pierced to access the ventral side of the diaphragm, which was also punctured. The thorax was dissected bilaterally along the lateral ribs in an inferior-to-superior direction to expose the beating heart. Slits were made at the bottom of the left ventricle and the top of the right atrium using fine scissors. A 24-gauge round-tipped feeding needle was inserted through the left ventricle into the proximal aorta and clamped in place. PBS (40 mL) was flushed through the rodent’s arterial system with cardiac facilitation, followed by 40 mL of 10% neutral-buffered formalin (VWA, Radnor, PA, USA) to fix the tissues. The brain was then removed and stored in formalin for histopathological analysis.

### 2.7 Histology

Brains collected upon transcardiac perfusion were transferred to 70% ethanol, cut, and placed into their corresponding labeled blocks, with more sections being cut when a gross inspection revealed a tumor. The tissues were then embedded in paraffin before being sectioned onto slides and stained using H&E. Immunostaining was performed using antibodies against von Willebrand factor VIII and Ki67. After incubation with these antibodies, an automated DAKO autostainer (Agilent, Santa Clara, CA, USA) was used to complete the immunostaining procedure. For H&E-stained slides, the HALO Image Analysis Platform was used for tumor area quantification. Using this platform, a random forest classifier was created to differentiate and quantify the tumor and normal brain tissue areas. For von Willebrand factor VIII- and Ki67-stained slides, the Aperio ImageScope platform was used for brown staining quantification. Aperio ImageScope utilizes a positive pixel count brown versus blue algorithm that quantifies brown staining compared to blue staining. For Ki67 analysis, the whole tumor was considered (200× magnification on Aperio), whereas only three hotspot areas were analyzed to quantify von Willebrand factor VIII staining. Since von Willebrand factor VIII staining is found in the endothelial layer of blood vessels, there was a reduced brown staining area compared to the whole tumor tissue area. By analyzing high-staining areas, the brown staining was more likely to reflect the staining intensity.

### 2.8 Statistical analysis

For imaging comparison, two-tailed Student’s *t*-tests were performed, with significance set at P < 0.05 with a minimum of 5 samples in each comparison. For Kaplan–Meier curve analysis, log-rank tests were performed, with significance set at P < 0.05 and with a minimum of 5 samples. All statistical analyses were performed in Microsoft Excel 2019 (Microsoft Corporation, Redmond, Wa).

## 3 Results

### 3.1 Study design

**Figure 1:**
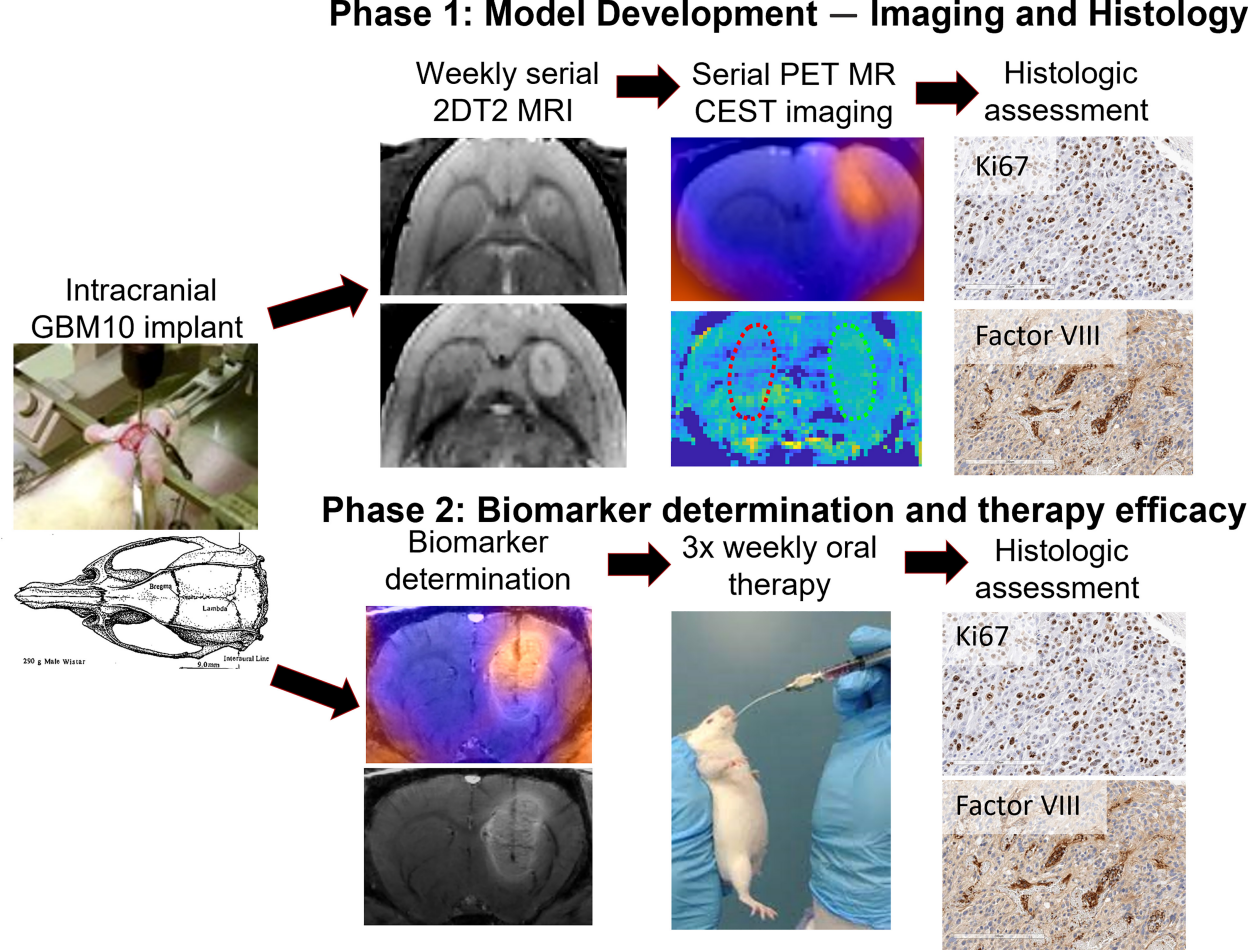
Summary of the study design. In the first phase, five rats were intracranially implanted with GBM10 cells and serially imaged initially with a T2 MRI sequence for monitoring lesion growth. Once lesion growth was confirmed, the rats underwent serial ^18^F-FET PET/CEST MRI on days 42 and 49 post-implantation. Once near-death endpoint criteria were reached, the brains were analyzed immunohistochemically for cellular proliferation (Ki67) and neovascularity (von Willebrand factor VIII). In the second phase, 15 rats were intracranially implanted with GBM10 cells, and on day 49, they underwent imaging for correlation analysis between ^18^F-FET PET and CEST MRI. Following imaging biomarker determination, the rats were then selected for similar tumor size across three groups, and oral therapy was administered as follows: vehicle, TMZ alone, or TMZ + idasanutlin. The rats received oral treatment three times weekly until near-death endpoint criteria were reached and were sacrificed for histological assessment of therapy response.

**Figure 2:**
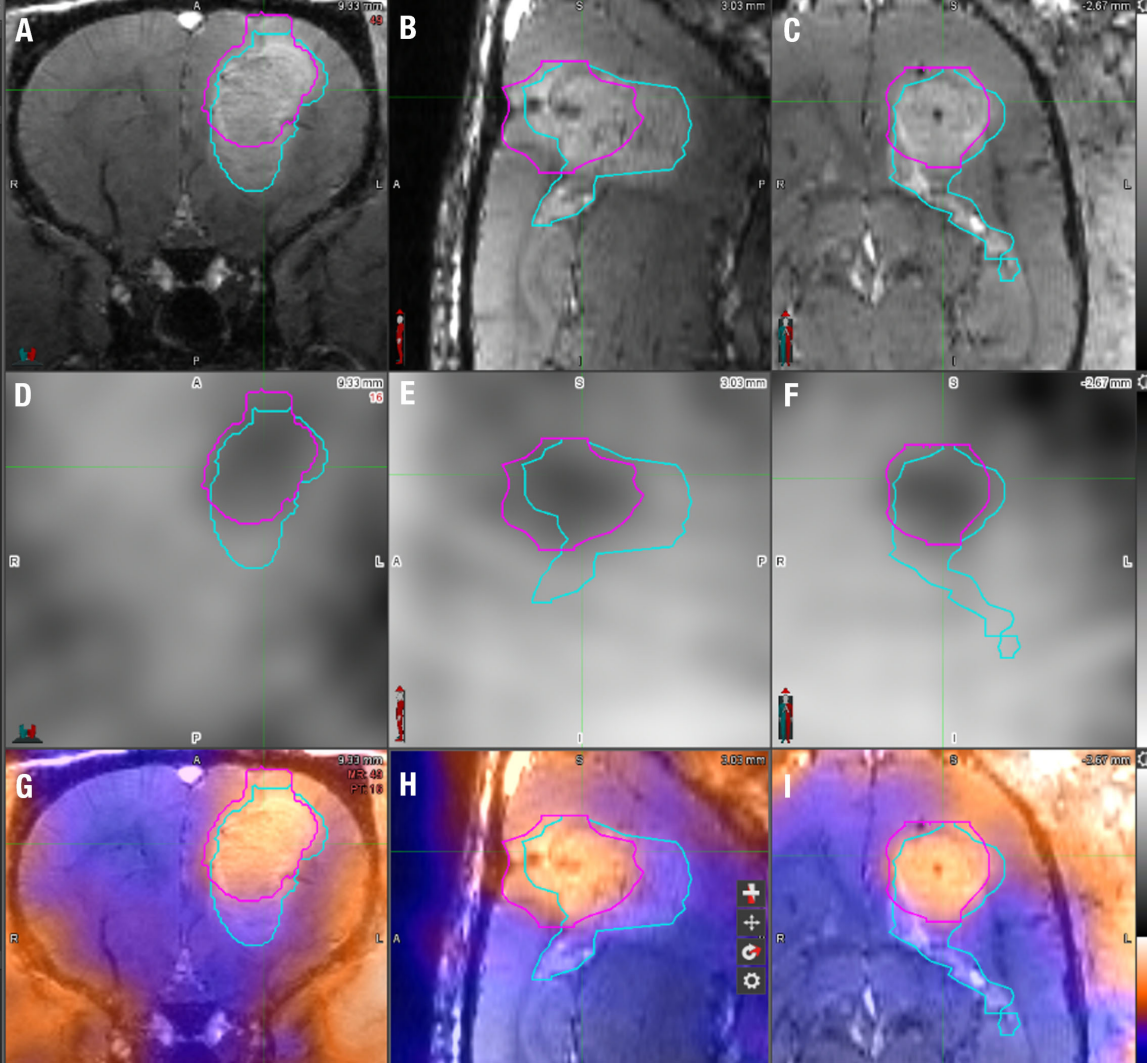
^18^F-FET PET/MRI analysis technique using MIM7 software. **(A–C)** The upper row displays the T1 FLASH post-contrast imaging in coronal, sagittal, and horizontal planes from left to right. **(D–F)** The middle row displays ^18^F-FET PET images of the same animal in the same coronal, sagittal, and horizontal planes. **(G–I)** The bottom row displays the fusion of the T1 FLASH contrast-enhanced sequence and ^18^F-FET PET image for the same animal. The light blue line represents the manual VOI drawn around the contrast-enhanced portion of the image around the tumor, and the pink VOI represents the ^18^F-FET PET activity, each superimposed on the images. The tumor volume was derived from the MRI VOI, while the % of the injected dose/mL (%ID/cc) and tumor-to-brain ratio (TBR) were derived from the ^18^F-FET PET VOI.

Initially, five *Rag2*-null rats were implanted with GBM10 cells and were allowed to recover with daily monitoring (**Figure 1**). All rats were observed daily following surgery until death using a standardized scoring system. The rats were serially imaged at days 21, 35, 42, and 49 after implantation to determine tumor growth *in vivo*. On days 21 and 35, all animals underwent two-dimensional (2D) T2-weighted MRI, and on days 42 and 49, they underwent hybrid ^18^F-FET PET/CEST MRI. The ^18^F-FET PET imaging was analyzed with an example shown in **Figure 2**, and the CEST MRI was analyzed with an example shown in **Figure 4F**. After animals reached near-death end point criteria, the brains were harvested following transcardiac perfusion. They were analyzed histologically using hematoxylin and eosin (H&E) staining as well as immunohistochemical staining for nuclear proliferation (Ki67) and neovascularization (von Willebrand factor VIII).

Using the information from phase I, a second set of *Rag2*-null rats (n = 15) were implanted with GBM cells and screened for consistent lesion growth with T2 MRI serially. On day 49 post-implantation, the animals underwent a hybrid ^18^F-FET PET/CEST MRI, which was then analyzed. Following *in vivo* tumor viability determination, the rats were then selected for similar tumor size across three groups, and oral therapy was administered as follows: saline (control; n = 5), TMZ alone (n = 5), and TMZ + idasanutlin (n = 5). The rats received oral treatment three times weekly until near-death endpoint criteria were reached. At that point, the brains were harvested and prepared for histological assessment, as explained above.

### 3.2 Phase 1: Model development—imaging and histology

#### 3.2.1 Initial serial MRI for GBM10 tumor growth assessment

**Figure 3:**
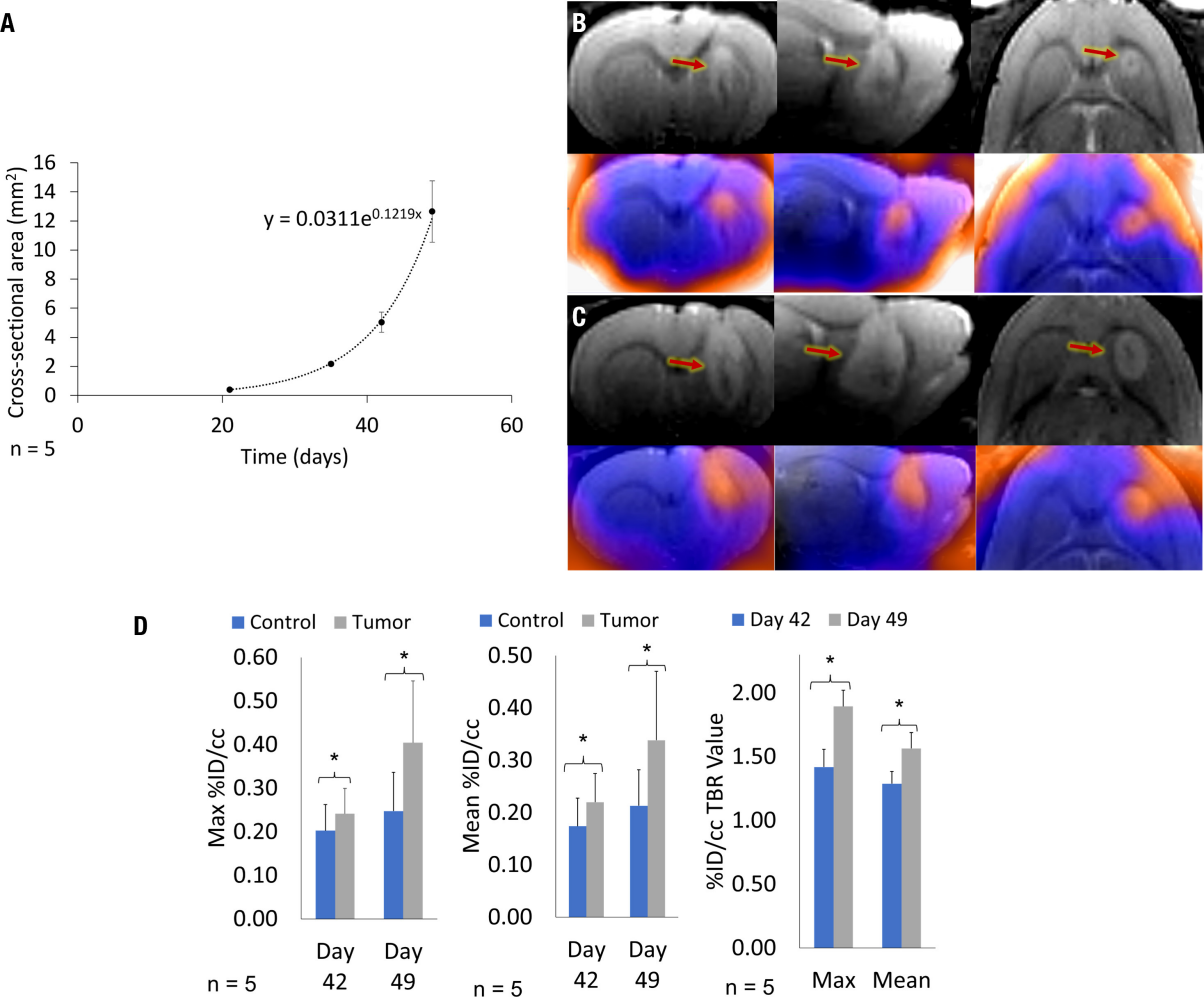
Serial MRI of a representative rat and cumulative mean GBM10 growth over time, following intracranial implantation based on serial ^18^F-FET PET/MRI. There is a marked increase in tumor size and a larger area of ^18^F-FET PET activity. **(A)** On day 49, the tumor was well delineated. The mean cumulative tumor growth over time was determined across all five *Rag2*-null rats, following intracranial GBM10 implantation assessed using serial MRI. **(B)** Triplanar coronal, sagittal, and horizontal 3D T2-weighted images (top row) and ^18^F-FET PET/MRI fusion (bottom row) were acquired simultaneously on day 42, showing tumor size and ^18^F-FET PET activity. **(C)** Triplanar coronal, sagittal, and horizontal 3D T2-weighted images (top row) and ^18^F-FET PET/MRI fusion (bottom row) were acquired simultaneously on day 49, showing tumor size and ^18^F-FET PET activity. **(D)** Quantification of serial Max and Mean %ID/cc, and Max and Mean %ID/cc TBR for serial ^18^F-FET PET/CEST MRI. Graphs present ^18^F-FET PET activity for both control (blue) and tumor (gray) VOI assessment, with significance at P < 0.05. There was a significant difference in ^18^F-FET PET activity in the tumor, based on Mean %ID/cc TBR and Max %ID/cc TBR, between days 42 and 49.

GBM10 tumor characteristics were assessed as hyperintensity on T2 sequences relative to the surrounding normal brain tissue signal in all five rats subjected to intracranial implantation using serial (21, 35, 42, and 49 days post-implantation) MRI. The tumor was well visualized within the brain parenchyma of the striatum and around the injection tract by day 35 in all rats, growing consistently in a tri-directional manner throughout the imaging studies. The mean tumor size based on the cross-sectional area on the coronal images was 0.5 ± 0.2 mm^2^ on day 21, 2.2 ± 0.9 mm^2^ on day 35, 5.2 ± 1.9 mm^2^ on day 42, and 12.5 ± 3.8 mm^2^ on day 49 (**Figure 3A**). There was a developing mass effect, but no significant midline shift or herniation was observed on day 49 after achieving a standard tumor size.

#### 3.2.2 Serial ^18^F-FET PET/CEST MRI during the early exponential phase of tumor growth

**Figure 4:**
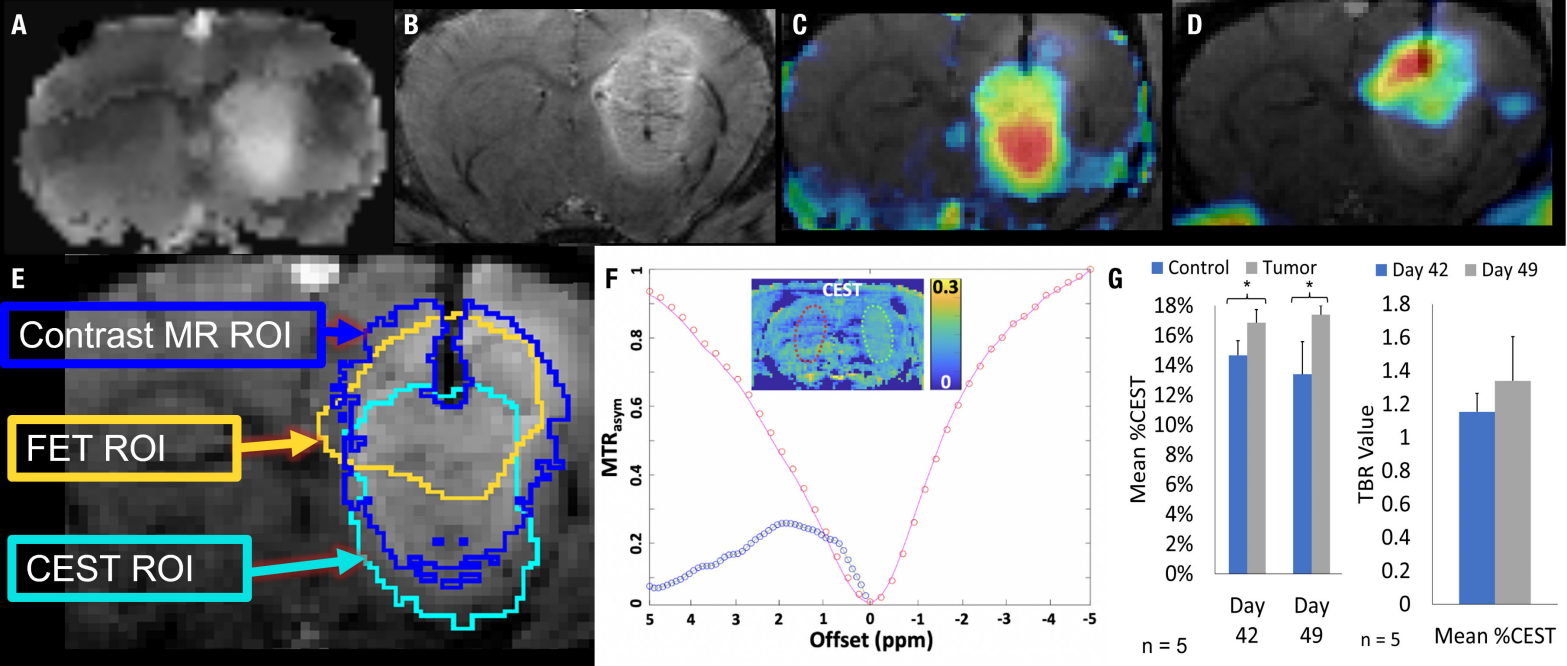
^18^F-FET PET/CEST MRI of a representative *Rag2*-null rat implanted with GBM10, using 3D T1 post-contrast enhancement and CEST MRI and ^18^F-FET PET. **(A)** Coronal view showing a region of signal hyperintensity in the left frontal lobe on a T2-CEST MRI. **(B)** Coronal view showing a large region of abnormal signal hyperintensity in the left frontal lobe on post-contrast 3D T1 MRI. **(C)** CEST MRI color map superimposed on the contrasted MRI showing higher intensity (red) in the lower half of the lesion and lower intensity (yellow and blue) in a non-overlapping manner compared with the contrast-enhancing signal in **(B, D)** ^18^F-FET PET color map superimposed over the contrast-enhanced MRI showing higher activity in the upper right-hand corner of the lesion (red and orange) with lower activity involving the upper half of the lesion. **(E)** The overlap of the ^18^F-FET, CEST, and post-contrast T1 ROIs. While there is a slight overlap in all three ROIs, each region is centered differently within the lesion, seen prominently in the differences in areas of high activity (red) seen in **(C, D, F)** The Z-spectrum, MTR_asym_, and amide-CEST MRI map of a GBM10 tumor-bearing rat brain are also shown with a 2D control region shown in red and an area of high CEST signal in the region of interest in green. **(G)** Simultaneously to the serial ^18^F-FET PET imaging, five animals were evaluated using serial CEST MRI on days 42 and 49 after implantation. The Mean %CEST of the tumor regions was significantly elevated (*P < 0.05) relative to control regions on both day 42 and day 49. However, there was no significant difference in the Mean %CEST of the tumor between days 42 or 49 and no difference in the Mean %CEST TBR between days 42 and 49.

A subset of five rats intracranially implanted with GBM10 was imaged using serial ^18^F-FET PET/CEST MRI on days 42 and 49 post-implantation for optimization of tumor evaluation in the early phase of exponential tumor growth. The goal was to determine when advanced neuroimaging should be implemented in this rat model and the tumor type to ensure adequate, discernible tumor activity above the background. Although the tumor was perceptible on both ^18^F-FET PET and MRI on day 42 (**Figure 3B**), all imaging characteristics were more robust on day 49 (**Figure 3C**). For this serial study, the mean MRI volume was determined based on a manually drawn VOI. The average MRI tumor volume was 12.4 ± 4.4 µL on day 42 and 25.2 ± 6.3 µL on day 49. However, contrast enhancement or increased T2 signal is considered nonspecific in clinical imaging, in which molecular imaging can play an important role. The contralateral control region was mirrored and had a comparable volume, with no significant difference in size compared to the tumor VOI. **Figure 3D** presents the values for Mean %ID/cc, Max %ID/cc, Mean %ID/cc TBR, and Max %ID/cc TBR, as well as the % increase from day 42 to day 49 and corresponding significance. The CEST MRI results as Mean %CEST and %CEST TBR are presented in **Figure 4G**. **Figures 4A–D** show examples of the lesion on the left and contralateral control brain on the right using a coronal 2D T2 sequence, 3DT1 Post-contrast, ^18^F-FET PET heat map fusion with MRI, and CEST MR heat map fusion with MRI. **Figure 4E** illustrates the different overlapping signals from **Figures 4B–D** *via* ROI comparisons (labeled dark blue for contrast MR ROI, yellow for FET ROI, and aqua blue for CEST ROI). Note how the different signals overlapped, but there were also qualitative visual differences in the “hot spot” activity areas for ^18^F-FET and CEST MR relative to each other and relative to the post-contrast gadolinium signal, suggesting different biological properties displayed within the tumor in different locations.

#### 3.2.3 Histological assessment of GBM10 tumor for human GBM-like characteristics

**Figure 5:**
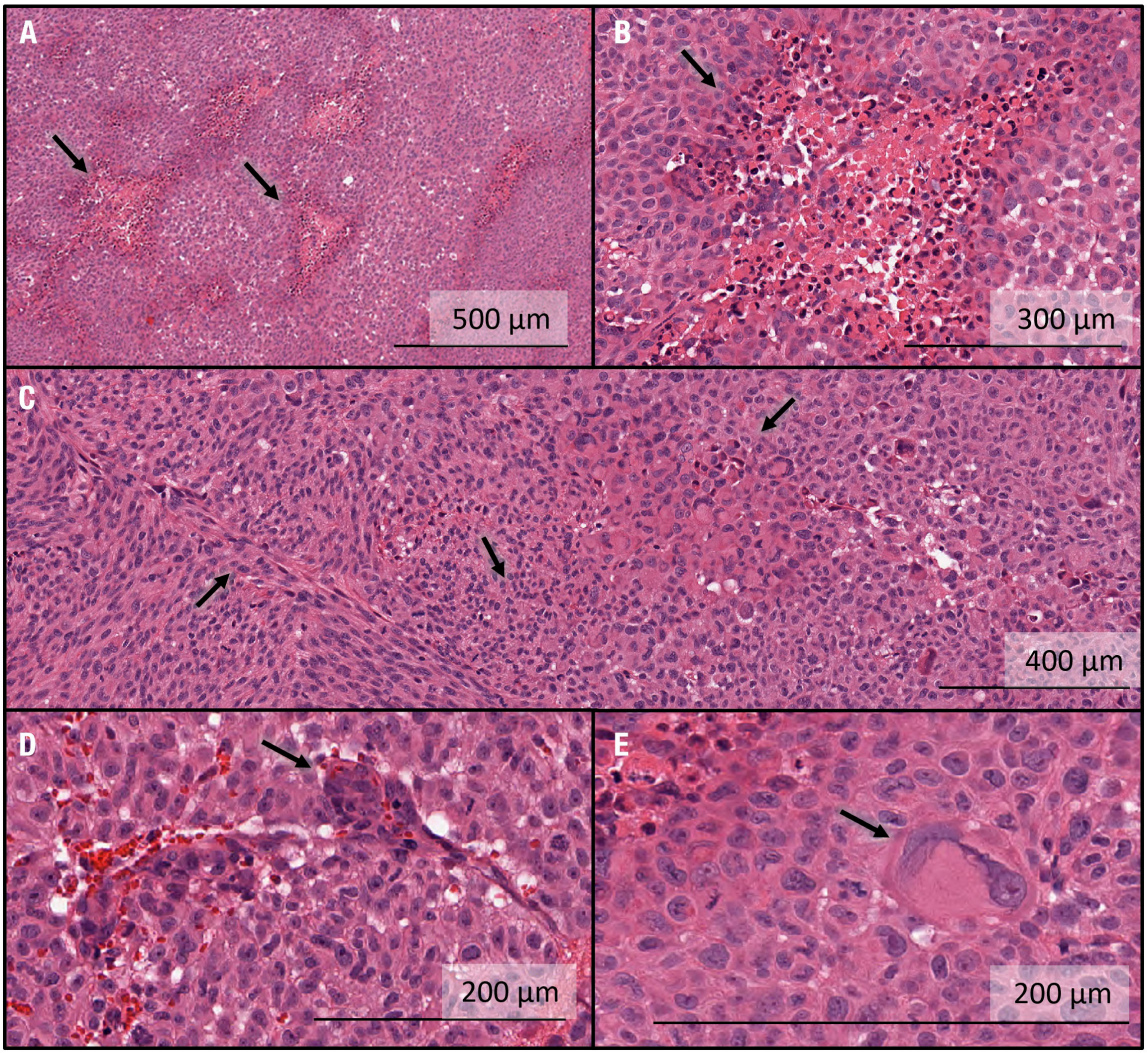
Histological characterization of intracranial GBM10 tumors in *Rag2*-null rats, based on H&E staining. **(A)** Multiple regions show high-density cell networks surrounding a centrally pink-stained area, indicating a rudimentary version of pseudopalisading with necrosis characteristic of human GBM tissue. **(B)** High-resolution image showing pseudopalisading with necrosis. The lighter pink areas with lower cell density show some degree of necrosis and are surrounded by cells with darker nuclei, again showing rudimentary pseudopalisading with necrosis. The morphology is not exactly similar to that seen in humans, where the tumors can grow for much longer than in rats. **(C)** Examples of an array of marked cellularity with hyperchromatism and pleomorphism. Note the variety of cell morphologies, from flat mesenchymal tumor cells (left-hand side) to giant anaplastic cells (right-hand side) and mitotic figures (middle). **(D)** Example of endothelial proliferation, another important feature of GBM. Glomeruloid vessel formation and endothelial multilayering secondary to endothelial hyperplasia are shown here. These changes are related to the tumoral secretion of vascular endothelial growth factor to overcome hypoxic conditions. These examples were less numerous here than typically seen in human GBM tissue. **(E)** Multinucleate giant cells with bizarre hyperchromatic nuclei are referred to as giant cell astrocytoma and present in some forms of human GBM.

All five tumor-bearing brains were sectioned and stained using H&E. Representative characteristics of the tumors in this group are shown in **Figure 5**. In all specimens, multiple regions were surrounded by a high-density network of cells with centrally pink staining, indicating a rudimentary version of pseudopalisading with necrosis characteristic of human GBM tissue (**Figures 5A, B**). The tumor morphology seen histologically was not exactly the same as in humans, where tumors can grow for much longer than in rats. Additional examples of an array of marked cellularity with hyperchromatism and pleomorphism were visible throughout the tumors (**Figure 5C**). Although not as frequent as in human GBM tissues, endothelial proliferation, another important feature of GBM, was visualized (**Figure 5D**). In addition, Glomeruloid vessel formation and endothelial multilayering secondary to endothelial hyperplasia were observed in selected regions (**Figure 5D**). Finally, multinucleate giant cells with bizarre hyperchromatic nuclei were also visualized, referred to as giant cell astrocytoma, and present in some forms of GBM (**Figure 5E**).

#### 3.2.4 Semi-quantitative evaluation of neovascularity and nuclear proliferation

**Figure 6:**
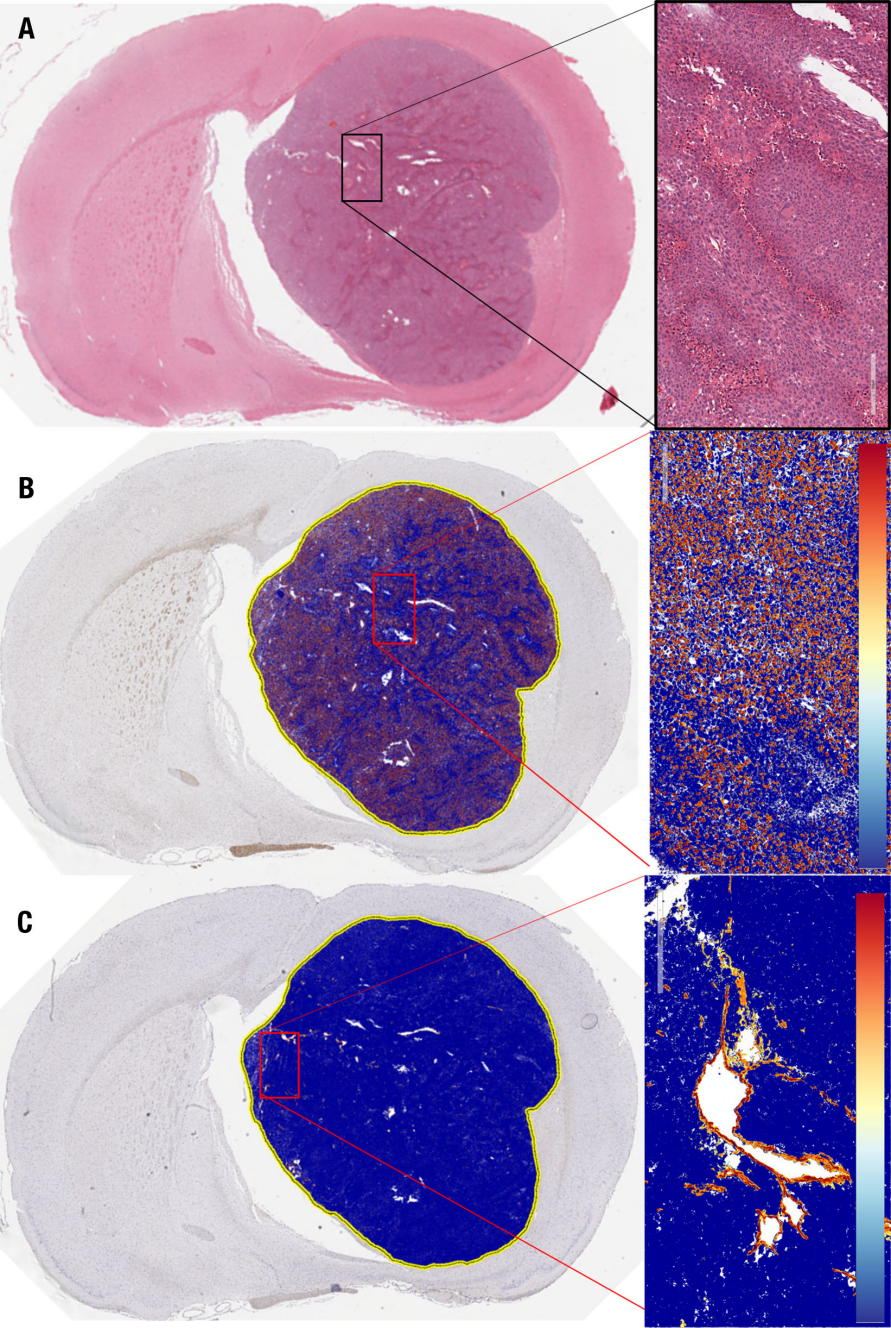
Histological analysis of GBM10 proliferation index and microvascular proliferation in a representative *Rag2*-null rat. **(A)** Coronal cross-section of the tumor, with a higher-resolution image of a region within the tumor (insert) demonstrating the high degree of cellularity and vascularity. **(B)** Ki67 staining of the same tumor as in **(A)**, with a higher-resolution image of a region within the tumor (insert). **(C)** von Willebrand factor VIII staining of the same tumor as in **(A, B)**, with a higher-resolution image of a region within the tumor (insert).

Additional histological characterization was performed to assess nuclear proliferation through immunohistochemical Ki67 staining and to assess neovascularity through immunohistochemical staining of Von Willebrand Factor VIII. A representative histological specimen from a single rat using H&E staining is depicted in **Figure 6A**. Ki-67 and Factor VII staining were semi-quantitatively analyzed using the Aperio software. A high percentage of proliferating cells was found in all Ki67-assessed brains, with a mean positivity of 34.1 ± 1.9%, ranging from 31.6 to 36.4% (n = 5) (**Figure 6B**). Analysis of von Willebrand factor VIII staining revealed a positivity of 3.5 ± 0.9%, ranging from 2.1 to 5.0% (n = 5) (**Figure 6C**).

### 3.3 Phase 2: Tumor viability determination at day 49 and subsequent therapy efficacy

#### 3.3.1 Correlation analysis between ^18^F-FET PET and %CEST

**Figure 7:**
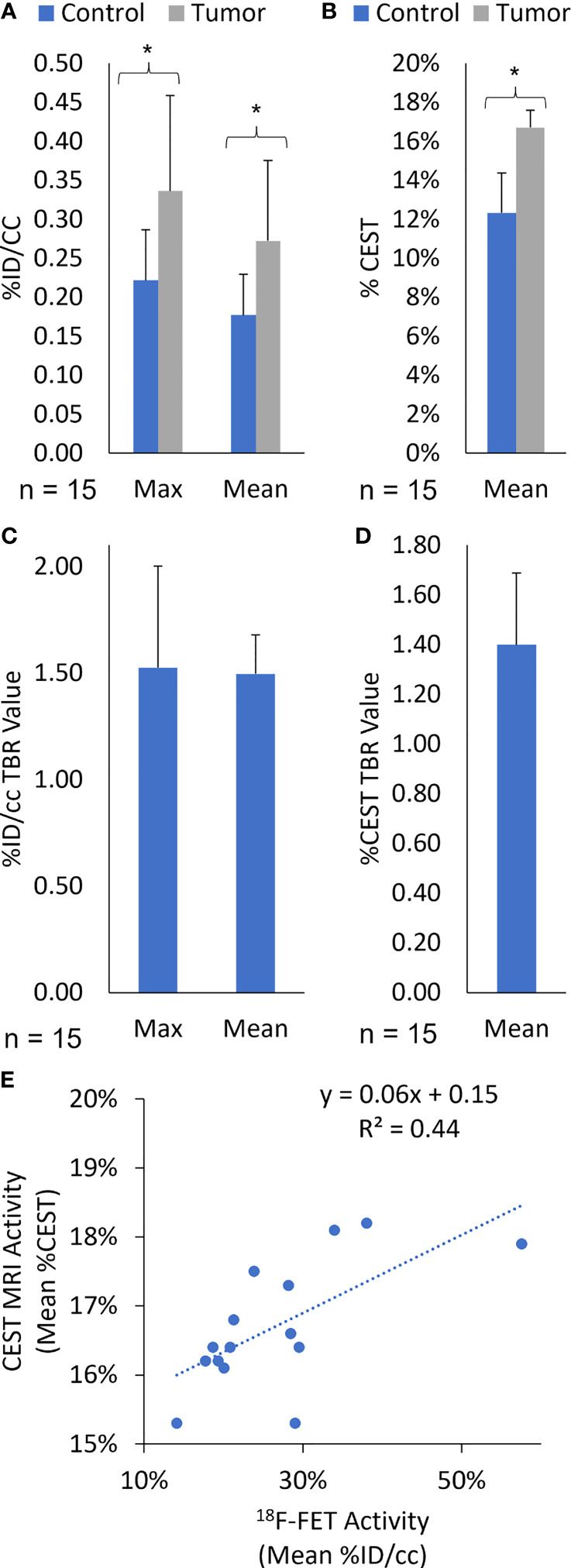
(Quantification of **(A)** Max and Mean %ID/cc, **(B)** Mean %CEST, **(C)** Max and Mean %ID/cc TBR, and **(D)** Mean %CEST TBR for ^18^F-FET PET/MRI on day 49 post-implantation. All *Rag2*-null rats intracranially implanted with GBM10 were imaged using high-resolution fusion ^18^F-FET PET/CEST MRI to evaluate tumor growth *in vivo*. The Mean %ID/cc, Max %ID/cc, and Mean %CEST were significantly higher in the tumor regions (*P < 0.05) than in the control regions of the brain. **(E)** Correlation of amide-CEST MRI activity and ^18^F-FET PET activity. The Mean %CEST obtained during CEST MRI was compared with the Mean %ID/cc obtained during the simultaneous ^18^F-FET PET acquisition for each animal at day 49 post-implantation (n = 15). The values were then subjected to linear regression analysis (r^2^ = 0.44).

In this study, the fifteen rats with lesions confirmed in the exponential growth phase on day 49 were imaged with simultaneous ^18^F-FET PET/CEST MRI. The average MRI tumor volume was 28.9 ± 11.9 µL, and the average ^18^F-FET PET volume was 27.2 ± 9.3 µL. Additional ^18^F-FET PET activity assessment included Mean %ID/cc, Max %ID/cc, Mean %ID/cc TBR, and Max %ID/cc TBR for imaging on day 49, with the significance of differences between control and tumor presented in **Figures 7A–D**. Linear regression analysis showed a correlation between Mean %ID/cc of ^18^F-FET and Mean %CEST (r^2^ = 0.44; **Figure 7E**). There was no correlation between ^18^F-FET Max %ID/cc activity and tumor volume (R^2^ = 0.009, P = 0.75), ^18^F-FET Mean %ID/cc and tumor volume (R^2^ = 0.02, P = 0.62), or %CEST activity and tumor volume (R2 = 0.04, P = 0.88) (**Figure 7E**).

#### 3.3.2 Therapy efficacy determination in the GBM10 *Rag2*-null rat model

**Figure 8:**
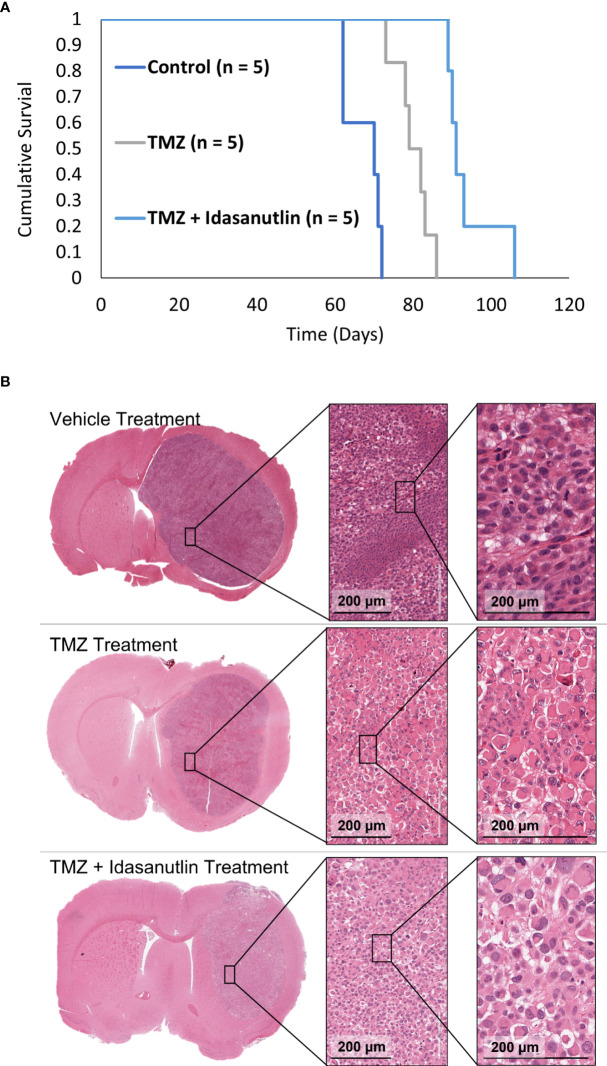
(Kaplan–Meier survival curves for control and chemotherapy-treated animals and Histological Characterization. **(A)** Cumulative survival was plotted against time for each group: control, TMZ-treated, and combination therapy-treated groups. The control (n = 5), TMZ-treated (n = 5), and TMZ + idasanutlin-treated (n = 5) rats implanted with GBM10 survived on average for 70, 79, and 91 days, respectively. Cumulative survival times differed significantly between groups (P < 0.001). **(B)** Top row: Coronal cross-section of the tumor of a representative control (vehicle-treated) rat, with higher-resolution images of a region within the tumor (inserts) showing high cellular density. Middle row: Coronal cross-section of the tumor of a representative TMZ-treated rat, with higher-resolution images of a region within the tumor (inserts) displaying intermediate cellular density and small pockets of cytoplasmic vacuolation. Although not widespread, this vacuolation was more frequent in the TMZ-treated group than in the control group. Bottom row: Coronal cross-section of the tumor of a representative TMZ + idasanutlin-treated rat, with higher-resolution images of a region within the tumor (inserts) showing low cellular density as well as large pockets of cytoplasmic vacuolation. Vacuolation was more consistently present in tumors of this treatment group, indicating a higher rate of apoptosis and necrosis in this group close to the experiment’s endpoint than in the control and TMZ-treated groups.

Following imaging characterization and confirmation of viable, treatable tumors, the rats were separated into three treatment groups: vehicle (control; n = 5), TMZ alone (n = 5), and TMZ combined with idasanutlin (n = 5). Cumulative survival was plotted over time (**Figure 8A**), with rats being sacrificed once near-death endpoint criteria were reached. During treatment, the rats were monitored thrice weekly for behavioral alterations. No significant visible behavioral side effects required excluding rats from the study. The average survival time of the vehicle-treated group was 67 ± 4 days, while that of the TMZ-treated group was 80 ± 4 days. The TMZ + idasanutlin-treated group survived an average of 94 ± 6 days. The TMZ-treated group survived significantly longer than the control group, and the TMZ + idasanutlin-treated group significantly out survived both the control and TMZ-treated groups (P < 0.001, log-rank test).

#### 3.3.3 Qualitative assessment of cell shrinkage in treated brain tumors

Vehicle-treated animals (n = 5) showed a nuclear proliferation positivity of 30.3 ± 4.9% and a neovascularity positivity of 3.2 ± 0.9%. These results were not significantly different from those of the model development histology (P > 0.05). In the TMZ- and TMZ + idasanutlin-treated groups, nuclear proliferation was also high, with a mean of 30.2 ± 2.9% (n = 5) and 26.5 ± 12.8% (n = 5), respectively.

However, a higher number of shrinking tumor cells was visible throughout the tumors (**Figure 8B**) as an indication of cellular apoptosis and suggestive of cells responding to the treatment (58). The number of vacuolated dying cells was visibly higher in the TMZ + idasanutlin-treated group than in the TMZ-treated group. In some specimens, this feature was intermixed with areas similar to those with high cellularity in the control brains.

Finally, neovascularization was semi-quantitatively assessed using the Aperio software, with a mean von Willebrand factor VIII staining positivity of 6.6 ± 0.9% in the TMZ-treated animals (n = 5) and 5.3 ± 1.5% in the TMZ + idasanutlin-treated group (n = 5). Images of representative brain specimens stained using H&E and for Ki67 and von Willebrand factor VIII are depicted in **Figures 6A–C**.

## 4 Discussion

This study is the first to develop a recurrent, drug-resistant patient-derived tumor model (GBM10) in SD *Rag2*-*Rag2* ^tm1Hera^ knockout rats showing similar histological/pathological features to GBM found in humans, which is an important initial step toward addressing the need for a humanized intracranial PDX rat model of GBM. The inclusion of serial brain MRI coupled with PET (PET/MRI) using a 9.4 Tesla system allowed the careful study of GBM growth characteristics, especially during the exponential growth phase of the tumor (post-implantation days 42–49). The correlation between Mean %ID/cc of ^18^F-FET PET activity and %CEST activity adds to the literature regarding the complementary nature of hybrid ^18^F-FET PET/CEST MRI in brain tumor management. For animal model validation, TMZ was tested against its vehicle since the former is the only standard-of-care chemotherapy agent that has been shown to be effective in GBM treatment. Combination therapy (TMZ + idasanutlin)-treated rats showed statistically significant prolongation of their survival time relative to those treated with TMZ alone, supporting the investigation of other combination therapies using this model.

The *Rag2*-null rat developed by Noto etal. (20) and used in this study was improved from prior rat models because it lacks mature B-cells and has severely reduced T-cell numbers relative to a normal SD rat (20). In our study, the presence of natural killer (NK)-cells in the model did not appear to affect the success rate of intracranially xenografting GBM10, although this could be further tested in the newly developed *Rag2*/*Il2rg* double-knockout rat, which is devoid of B-, T-, and NK-cells (18). While the *Rag2*-null model is permissive to different flank-implanted cancer cell lines, including glioma-derived U87MG cells, the more specific intracranial implantation of cancer cells has not yet been characterized. Although there are many different immunodeficient mouse models, few successful immunodeficient rat models exist. The most well-known immunodeficient rat model is the National Institute of Health (NIH) nude (RNU; NIH-*Fox1* ^rnu^) rat, which is devoid of T-cells but has a normal B- and NK-cell repertoire (59). Therefore, human tumor cell engraftment in this model has been limited, especially because the NIH nude rat develops some degree of immunocompetence as it ages (60–62). In a preliminary unpublished study from our lab, only 1 of 10 (10%) NIH nude rats successfully grew GBM10 tumors, compared with 16 of 18 (94%) *Rag2*-null rats using the same implantation technique (data not shown). In another preliminary unpublished study from our lab, four of four (100%) *Rag2*-null rats were permissive to intracranial U87 tumor growth. Other reasons may explain the low intracranial tumor growth success rate of GBM10 in NIH nude rats, and further experiments were not performed to confirm our initial findings. Therefore, definitive conclusions cannot be drawn regarding the ability of NIH nude rats to grow GBM10 tumors using only our preliminary data. Nevertheless, the high GBM10 implantation success rate in our *Rag2*-null rat cohort is promising, and the tumor growth characteristics were relatively consistent across all rats studied.

Histological analysis of the GBM10 tumors revealed morphological characteristics consistent with those of human GBM, including high nuclear atypia and cellular proliferation, nuclear palisading, pseudopalisading necrosis, and microvascular proliferation. In this study, Ki67 staining, a standard method for calculating the percentage of nuclear proliferation in human GBM, was consistent with that observed in patient GBM histological analysis, representing a high-grade tumor. However, the well-defined margins of the tumor (i.e., lack of infiltration/individual cell invasion) and lower-than-expected microvascular proliferation need to be further studied. These limitations could likely be overcome by using lower passage rates from the parent tumor tissue through neurosphere culture or more direct tumor fragments following human biopsy or tumor resection. Nevertheless, these techniques are complex and expensive. The success of cell implantation following limited monolayer passages supports experimenting with other more sophisticated options for xenotransplantation.

Our next objective was to characterize the tumor growth rate characteristics *in vivo* by utilizing clinically relevant, noninvasive neuroimaging modalities at both the early and late stages of tumor growth. In the early tumor growth phase (i.e., days 21 and 35 post-implantation), a simple 2D T2 sequence was successfully employed in the coronal plane as an economical means to establish evidence of increasing tumor volume over time. Serial ^18^F-FET PET/CEST MRI was then performed on days 42 and 49 post-implantation in a small cohort of rats for advanced dual-modality tumor detection and molecular characterization. There was a noticeable increase in tumor size, ^18^F-FET activity, and CEST MRI activity over the course of a single week during the exponential tumor growth phase. Besides the visibly larger tumor on day 49 than on day 42, many imaging parameters showed a significant difference between these two days, except for the PET TBR. The primary issue is likely related to the high variability in the signal across both the tumor and control regions, which are accounted for to some degree when a TBR is utilized. This is consistent with the clinical reporting of ^18^F-FET, where the TBR is the international standardized metric for brain tumor reporting for this PET radiotracer (63, 64). Therefore, *in vivo* ^18^F-FET PET for pre- and post-therapy assessments may not be as useful over the course of a single week due to its variability. At the same time, CEST MRI might provide an advantage to earlier detection since it does not appear to suffer from the same degree of variability. Pre- and post-therapy imaging assessments may be better performed over at least 2–3 weeks following therapy and allow for further tumor growth to detect a therapeutic response using this imaging paradigm.

^18^F-FET PET suffers from some degree of nonspecific uptake in the presence of a disrupted BBB. However, the TBR_max_ in clinical imaging has proven to be the most reliable indicator of viable tumors. As depicted visually in **Figures 2** and **4E**, the degree of contrast enhancement was often not completely congruent with the ^18^F-FET activity in terms of location, which is consistent with the findings in patients (65, 66). While this occurs in the clinic, it is also recognized as a potential source of differing tumor growth parameters in the experiments conducted herein. The TBR_max_ obtained in this study was overall lower (~1.9) than that reported in humans (>2.5) and in intracranially implanted murine GL261 GBM in mice (>2.5 in weeks 4 and 5 measured as standard uptake value_max_/background) (35). Future studies will histologically assess the expression of the LAT1 receptor in GBM10 cells following *in vivo* growth in *Rag2*-null rats. The initial characterization of our data using the standard uptake value was not as reliable as that using %ID/cc, likely because both males and females, which show significant differences in body weight, were included in this study. Nonetheless, in our cohort, there was a close-to-equal balance between males and females (n = 7 females, n = 8 males). Rather, the %ID/cc analysis yielded much better consistency across animals regarding data analysis. The influence of sex was studied to some extent, with ^18^F-FET uptake in females being, on average higher than that in males in both control and tumor VOIs, which is consistent with the differences found in clinical studies using ^18^F-FET PET imaging (67). Therefore, sex should also be carefully considered when assessing therapy using ^18^F-FET PET imaging.

In this study, a potentially clinically relevant imaging biomarker correlation was discovered between ^18^F-FET PET uptake using %ID/cc and %CEST activity on CEST MRI. This makes sense since both imaging approaches assess some elements of the cellular protein machinery (LAT1 receptor in the case of ^18^F-FET PET and intracellular mobile protein phase in the case of CEST MRI). Although the manner in which the CEST data was acquired in our study (2D in CEST MR vs. 3D in ^18^F-FET PET) did not permit comparison between CEST MRI and ^18^F-FET PET volumes, there was sufficient evidence in the fully co-registered fusion between post-contrast MRI, ^18^F-FET and CEST MRI activity map to show the feasibility of studying hot spot co-localization. This may partially explain why the two signals are correlated but not perfectly correlated. Future studies will benefit from higher sample size multiparametric mapping of intratumoral activity differences across ^18^F-FET PET and CEST MRI sequences acquired in the same animal. These findings are key for identifying very small (~1 mm) viable tumors against a background of tissue changes, with the potential to address a significant medical conundrum for neuroradiologists performing brain tumor imaging. To highlight this importance for clinical brain tumor management, two clinical studies have compared the role of amide-CEST MRI with that of amino acid PET imaging. In the first, Schön etal. (41) evaluated 46 newly diagnosed glioma cases and determined that the volume of the abnormal CEST MRI signal was overall larger than the ^18^F-FET PET signal, which was more discernible in GBM than in lower grade gliomas (41). In the second published clinical study, ^11^C-methionine, another well-characterized amino acid PET agent that utilizes the LAT receptor, was compared with amide-CEST MRI in 43 patients with gliomas in the post-treatment period to determine the diagnostic performance of the two modalities (68). Amide-CEST MRI appears to perform better than ^11^C-methionine in distinguishing recurrent disease in high-grade gliomas, although a rationale has been made for the continued use of multimodality imaging to cross-reference imaging properties for higher overall diagnostic accuracy (69). The findings of these studies are significant because they suggest that CEST MRI can better define the infiltration of GBM, in particular, and glioma, in general, in the tumor periphery. Moreover, the volume of overlap between the CEST MRI and ^18^F-FET or ^11^C-methionine PET activity obtained in these published studies provides additional evidence of a complementary role of the two imaging modalities when performed simultaneously, both measuring cellularity. As cancer therapy continues to evolve and offers a more personalized medicine centered on tumor biomarkers, the development of complementary PET and MRI agents hold great promise in pairing therapy with diagnostics (70).

Another objective of this pilot study was to assess the therapeutic response of intracranial GBM10 xenografts *in vivo*. TMZ treatment granted a significant survival advantage to rats relative to treatment with the vehicle (P < 0.001). This was unexpected because the tumor was derived from a patient whose disease recurred despite undergoing TMZ therapy. Our initial hypothesis was that TMZ would have no effect, but the average survival of rats was indeed extended by approximately 2 weeks relative to that of control animals. Furthermore, the combination of TMZ + idasanutlin further extended the survival of rats (P < 0.001) relative to that in both the TMZ-treated and control groups; on average, rats in the combination therapy group survived one more month than control rats.

Idasanutlin was chosen in combination with TMZ because it has great systemic exposure, is metabolically stable *in vivo* and non-genotoxic, and crosses the BBB (71–73). Idasanutlin was efficacious in our study, likely acting similarly to the MDM2 inhibitor nutlin 3a, which suppressed GBM10 xenografts in mice in combination with TMZ through activating the p53 pathway, downregulation of DNA repair proteins and continued DNA damage (14). However, therapy solely with idasanutlin may lead to secondary resistance since prolonged idasanutlin treatment might induce *de novo* resistant p53-mutated populations (74). Therefore, MDM2 inhibitors are more likely to be beneficial in combination with other agents that have non-overlapping mechanisms, especially if they can kill p53-mutated GBM cells (14).

While overall survival was assessed in the combination therapy group, serial assessment using ^18^F-FET PET/MRI for biomarker level determination was not performed before and after treatment, which will be the focus of future experiments. Histologically, there was a qualitative trend in the single- and dual-treatment groups showing a higher number of cells undergoing shrinkage and presenting small, fragmented nuclei, suggesting some degree of apoptosis was more evident in the combination therapy group. However, these results were difficult to quantify due to the relative heterogeneity within each treatment group. This suggests a mixed response, with the development of resistance to therapy.

Our study has several limitations. A major limitation is a high cost and difficult logistics of performing serial imaging using ^18^F-FET, which requires careful coordination during and after cyclotron production and limits the number of rats that can be imaged daily. PET/MRI is inherently expensive, limiting the generation of a large set of images more akin to a clinical brain tumor protocol, including diffusion- and perfusion-weighted imaging. Moreover, as only post-contrast T1 imaging was performed, signal hyperintensity was assumed to be the result of post-contrast enhancement and not intrinsic T1 hyperintensity, as can be seen when hemorrhage occurs. Limitations also arose from the dependence of MTR_asymm_ on saturation amplitude (75). A magnitude of 5 μT is reasonable for animal research systems since clinical scanners have specific absorption rate limitations (up to 4 μT) (76). In this study, we only used one saturation power, limiting our ability to explore its effect on the MTR_asymm_. Future studies could evaluate this effect in more depth. Additionally, the CEST MR images were obtained using a 2D rather than a 3D sequence, limiting the comparison to PET data. Because of this, no volumetric comparisons between PET and CEST data could be made. Now that the feasibility of studying MR-CEST in this animal model has been established, we intend to acquire 3D CEST volumetric tumor data in future studies in a manner that can be compared with the ^18^F-FET PET volumes (77–80) In addition, the emerging quasi-steady-state CEST reconstruction potentially aids the standardization of *in vivo* CEST image analysis, which can be adopted in future studies for quantitative CEST MRI (81–83). Another limitation that deserves consideration is the scarce availability of hybrid PET/MRI scanners for small animals, which means that the simultaneous acquisition of ^18^F-FET and CEST MRI data for animal model development will only be possible for a few investigators. Despite this, there is ongoing work to make PET/MRI scanners more accessible in addition to improving the specificity and efficiency of CEST acquisition and reconstruction to provide more metabolic information about tumor growth (84–88). Furthermore, the resolution of PET imaging is relatively low for tumors of only a few millimeters in size. The motion was another limitation encountered in this study that needed to be considered during analysis and likely contributed to the variation in results. Finally, although therapy could extend rats’ survival, its toxicity was not assessed, except for clinical behavioral observation.

The results of this study demonstrated good reproducibility and the establishment of a recurrent, drug-resistant, patient-derived GBM10 tumor in the recently developed *Rag2*-null rat. Histological characterization confirmed human GBM-like characteristics of the GBM10 tumor by studying nuclear atypia/proliferation and neovascularity patterns in a subset of rats. To our knowledge, intracranial GBM tumors have not been reported using this new *Rag2*-null transgenic rat strain, with our study providing a strong rationale for the implantation of patient-derived xenografts in this rat model. The model permitted in-depth investigation of disease progression in a complex *in vivo* environment and allowed noninvasive monitoring of tumor growth using clinically relevant cross-sectional imaging modalities. By performing ^18^F-FET PET and CEST MRI simultaneously, a correlation was determined between the tumor cell demand for amino acids and tumor intracellular mobile phase protein levels captured by the Mean %ID/cc of ^18^F-FET activity and Mean %CEST activity on MRI, respectively. Finally, the GBM10/*Rag2*-null rat model was validated for drug testing using single and combination therapy regimens, which was demonstrated to increase overall survival, supporting testing of other combination therapies in this animal model. The results of this study are an important first step in addressing the critical need for developing animal models that more closely mimic the therapy resistance of human GBM.

## Data availability statement

The raw data supporting the conclusions of this article will be made available by the authors, without undue reservation.

## Ethics statement

The animal study was reviewed and approved by the Indiana University School of Medicine Institutional Animal Care and Use Committee (Animal Use Protocol Number: 20118).

## Author contributions

LJ and MV contributed to the conception and design of the study, performed the experiments, organized the database, performed the statistical analysis, wrote the first draft of the manuscript, and revised subsequent versions. MM, Q-HZ and SS performed experiments. BS, GS, SM, GH, PS, and NW organized the database, performed the statistical analysis, and wrote sections of the manuscript. KP, HZ, and SD contributed to the conception and design of the study and wrote sections of the manuscript. EB contributed to the conception and design of the study and performed experiments. All authors contributed to the manuscript revision, read and approved the submitted version.

## Funding

This study is funded partly by the American Cancer Center Society Institutional Grant # 134125-IRG-19-144-34-IRG, the Indiana University Simon Comprehensive Cancer Center (IUSCCCC), and an award from the Indiana University School of Medicine (Indiana University School of Medicine Biomedical Research Grant).

## Acknowledgments

The following research cores are acknowledged: Dept. of Radiology and Imaging Sciences *In Vivo* Imaging Core, Dept. of Radiology and Imaging Sciences Molecular Imaging Ligand Development Program, IU Simon Comprehensive Cancer Center, and the IU SCCC *In Vivo* Therapeutics Core.

## Conflict of interest

The authors declare that the research was conducted in the absence of any commercial or financial relationships that could be construed as a potential conflict of interest.

## Publisher’s note

All claims expressed in this article are solely those of the authors and do not necessarily represent those of their affiliated organizations, or those of the publisher, the editors and the reviewers. Any product that may be evaluated in this article, or claim that may be made by its manufacturer, is not guaranteed or endorsed by the publisher.

## Abbreviations

2D, two-dimensional; 3D, three-dimensional; ^18^F-FET, ^18^F-fluoroethyl-*L*-tyrosine; BBB, blood-brain barrier; CEST, chemical exchange saturation transfer; GBM, glioblastoma; FOV, field of view; H&E, hematoxylin and eosin; LAT, L amino acid transporter; MAP, maximum *a posteriori*; MDM2, mouse double minute 2; MRI, magnetic resonance imaging; MTR_asym_, asymmetric magnetization transfer ratio; NIH, National Institute of Health; NK, natural killer; PBS, phosphate-buffered saline; PDX, patient-derived xenograft; PET, positron emission tomography; *Rag2*, recombination activating gene 2; RARE, rapid acquisition with refocused echoes; SD, Sprague–Dawley; TBR, tumor-to-brain ratio; TE, time-to-echo; TMZ, temozolomide; TE, time-to-echo; VOI, the volume of interest.
